# Joshua Frederick Parsons

**Published:** 1912-03

**Authors:** 


### JOSHUA FREDERICK PARSONS, M.R.C.S., L.S.A.

We have to record the death at his residence, Garston, Frome, of Mr. Joshua Frederick Parsons, M.R.C.S., L.S.A., on the 9th of February. He had been in failing health for some time, suffering from a heart lesion which followed after a serious attack of pneumonia in 1905.

Mr. Parson's professional career was spent entirely in Frome, where for most of the time he enjoyed an excellent private practice, and was Medical Officer of Health to the Urban District Council. He was the third son of the late Mr. Joshua Parsons, an able country doctor, who practised for many years in Beckington and afterwards in Frome, and brought up a large and highly-gifted family, including Dr. Franklin Parsons, late of the Medical Department of the Local

Government Board, and Mr. Alfred Parsons, R.A., the famous English landscape painter.

Mr. Frederick Parsons received his medical education at St. Mary's Hospital, London, where as contemporaries in his student days he had Sir Malcolm Morris and Mr. Edmund Owen, both of whom enthusiastically remember his great ability, Courageous, resourceful, with a clear-thinking head, he was in possession of every factor that made for success in medical practice.

He had qualified just at the time that abdominal surgery was making rapid strides, and having made the most of his opportunities as a student and resident at his hospital, he fostered the knowledge and skill he had acquired, and was the first in the part of the country he practised in to perform ovariotomies and other abdominal operations in the Cottage Hospital which his father and himself succeeded in establishing in Frome. In consequence of this he was well and widely known as a surgeon in his own town and the north-east part of Somerset. He was a good diagnostician and an excellent manipulator.

As a townsman of Frome Mr. Parsons was very popular, and universally liked. He was a keen sportsman, an excellent shot, and a first-rate billiard player. In his early days he was one of the best exponents of the Rugby game of football in the County of Somerset.

His social side was well developed. He was an excellent raconteur, his repertoire seemed never to be exhausted, nor did his stories lack freshness or variety.

